# Classification of trauma-related preventable death; protocol of a Delphi procedure

**DOI:** 10.1371/journal.pone.0298692

**Published:** 2024-05-06

**Authors:** Nadia A. G. Hakkenbrak, Annelieke M. K. Harmsen, Wietse P. Zuidema, Udo J. L. Reijnders, Patrick Schober, Frank W. Bloemers

**Affiliations:** 1 Trauma Unit, Department of Surgery, Amsterdam University Medical Centre, Amsterdam, The Netherlands; 2 Trauma Unit, Department of Surgery, Northwest Clinics, Alkmaar, The Netherlands; 3 Department of Forensic Medicine, Public Health Service of Amsterdam, Amsterdam, The Netherlands; 4 Department of Anesthesiology, Amsterdam University Medical Centre, Amsterdam, The Netherlands; Erasmus University Rotterdam, NETHERLANDS

## Abstract

**Background:**

Trauma-related (preventable) death is used to evaluate the management and quality of trauma care worldwide. Therefore, it is necessary to identify fatalities in the trauma care population and assess them on preventability. However, the definition on trauma-related preventable death lacks validity due to differences in terminology and classifications. This study aims to reach consensus on the definition of trauma-related preventable death by performing a Delphi procedure, thereby, improving the assessment of trauma-related preventable death and thereby enhancing the quality of trauma care.

**Methods:**

Based on the results of a recently performed systematic review Hakkenbrak (2021). The definitions used to describe trauma-related preventable death could be divided into four categories: 1) Clinical definition based on panel review or expert opinion, 2) Trauma prediction algorithm, 3) Clinical definition with an additional trauma prediction algorithm and 4) Others (e.g., errors in care or detailed clinical definition). A three round, electronic Delphi study will be performed in the Netherlands to reach consensus. Experts from the department of Trauma surgery, Neurosurgery, Forensic medicine, Anaesthesiology and Emergency medicine, of the designated Level 1 trauma centres in the Netherlands, will be invited to participate. In the first round the panel will comment on the composed categories and trauma prediction algorithms. In the second and third round a feedback report will be presented and the questions with disagreement will be retested.

**Discussion:**

The identification and assessment of trauma-related preventable death is necessary to evaluate and improve trauma care. Therefore, a valid, fair, and applicable definition of trauma-related preventable death is required. The Delphi technique is utilized to reach group consensus to obtain a scientifically valid definition of trauma-related preventable death.

## Background

Annually, trauma-related death accounts for over 140.000 fatalities within the European Union [1]. Trauma-related death is used to evaluate the management and quality of trauma care [2]. Therefore, data are collected and assessed on the preventability of trauma-related deaths [2]. Unfortunately, the definition of trauma-related preventable death (TRPD) lacks validity due to differences in the usage of terminology in literature. Definitions based on clinical evaluation, trauma prediction algorithms, errors in care or a combination of these are used randomly.

In order to facilitate the assessment of TRPD, aiming to improve quality of trauma care, a validated definition on TRPD should be constituted. Hence, a systematic review was performed to identify the most frequently used definitions of TRPD [3]. This review described four categories:

I) Clinical definition based on panel review or expert opinion.
II) Trauma prediction algorithm.
III) Clinical definition based on panel review or expert opinion with an additional trauma prediction algorithm.
IV) Others (e.g., errors in care or extended clinical definition)

The first category, clinical definition, contains three groups [3]:

1) TRPD describes fatalities where death could have been avoided or was caused directly by an avoidable error, delayed or suboptimal care.
2) Trauma-related potentially preventable death (TRPPD) describes fatalities in which death could have been avoided under optimal care conditions.
3) Trauma-related non-preventable death (TRNPD) describes fatalities where death was unavoidable due to the severity of the injuries or co-morbid factors despite optimal care conditions.

The second category identifies TRPD based on a trauma prediction algorithm. The most frequently used algorithms are the Injury Severity Score (ISS), Probability of survival (Ps) and Trauma Injury Severity Score (TRISS). Depending on the algorithm, the score is based on the mechanism of trauma, severity of the injuries, affected body region and clinical parameters such as the systolic blood pressure, Glasgow Coma Scale, and respiratory rate.

The third category combines both previously described categories [2]:

1) TRPD: injuries were considered survivable under optimal care, Ps >50% or ISS <20.
2) TRPPD: severe injuries but survivable under optimal care, Ps 25–50% or ISS 20–50.
3) TRNPD: non-survivable injuries, Ps <50% or ISS>50; 4) non-preventable death, but with care that could have been improved.

The final category (IV) includes the less frequently used classifications, such as errors in care or a clinical definition with a very detailed description of the injuries (Supplement 1 in S1 File).

Nearly 85% of the articles used a clinical definition (Category I, III, IV). In 41% an additional algorithm or extended clinical definition was used.

Consensus methods such as the Delphi and nominal group technique, also known as the expert panel, are commonly used in health care to create agreement amongst health care providers [4–6]. The Delphi procedure anonymously questions a group of experts about a subject during several rounds of enquiry [7]. Hereby, minimizing social pressure, enabling experts with different backgrounds and geographic locations to participate, whilst adjusting the design of the rounds of enquiry to the requirements of the research subject [7]. Based on the results of the previously elucidated systematic review the Delphi procedure was judged as most suitable to seek agreement and form consensus on the definition of TRPD by a multidisciplinary expert panel [3, 7]. The aim of this study is to present the design of the Delphi procedure on TRPD.

## Methods

The Delphi technique, initially developed in the 1950’s, is designed to assess complex dilemma’s that need to be addressed by a group of experts [5–8]. A structured approach is used to guide an anonymous dialogue to generate discussion and converge toward group consensus by iteration and controlled feedback [9, 10]. This technique is most suitable to create a validated, multidisciplinary supported definition of TRPD.

This protocol will provide information on the Delphi procedure including selection of the panel members, anonymity of the panelists, structure (controlled feedback, iterative rounds), consensus- and closing criteria, and analysis of the results [7, 10, 11]. This Delphi procedure will be conducted in accordance with the Conducting and Reporting Delphi Studies (CREDES) recommendations [7].

### Steering committee

The steering committee is composed of six individuals working in the field of critical care and forensic medicine. The committee, consisting of the authors, has prior experience in performing a Delphi procedure and will decide on the format of the questionnaires, data analysis, feedback documents and management of the Delphi process.

### Selection of the panel members

Based on the previously mentioned systematic review the following medical disciplines will be invited to participate in the panel [3]: 1) Trauma surgery, 2) Neurosurgery, 3) Forensic medicine, 4) Anesthesiology and 5) Emergency Medicine. The Netherlands encounters eleven Level 1 trauma centers [12]. A Level 1 trauma center, as described by the American College of Surgeons Committee on Trauma (ACSCOT), must have at least 1,200 trauma patient admissions or 240 admissions with an ISS of more than 15 per year, maintain a surgically directed critical care service including an attending trauma surgeon (present at the emergency department within 15 minutes upon request), participate in the training of residents, education and outreach activities and conduct trauma research [13]. The following assigned Level 1 and affiliated trauma centers will be contacted to participate: Amsterdam University Medical Center, Northwest Clinics, Elisabeth TweeSteden Hospital, Erasmus Medical Center, Haaglanden Medical Center, Haga Hospital, Isala, Leiden University Medical Center, Medical Spectrum Twente, Maastricht University Medical Center, Radboud University Medical Center, University Medical Center Groningen and University Medical Center Utrecht.

All medical secretaries of the eligible disciplines of the assigned hospitals will be contacted via phone to provide background information on the study and verify the email addresses. An invitation e-mail will be sent containing information on the aim of the study and a request to participate.

The literature remains unambiguous with regard to the standard size of the panel [6, 7]. A number close to 30–50 is considered adequate depending on the complexity of the problem, heterogeneity of the panel and resources [10]. In order to obtain a widely supported consensus amongst physicians the aim is for ten experts per discipline to complete the survey, resulting in at least 50 panelists. Additionally, it is preferred that one expert per discipline of each Level 1 trauma center participates. Whilst taking a response rate of up to 70–80% and a loss of 50% during the study period into account all practicing medical specialists of the eligible disciplines and centers were invited to participate [4]. If the number of 50 panelists is reached, with 10 experts per discipline, but not all Level 1 trauma centers are represented the missing centers will be reminded twice. If the number of experts is not met all centers will be reminded twice to participate, until the number of 50 panelists, with 10 experts per discipline, is reached. Overall, there will not be sought for parity in gender, ethnicity, or seniority levels amongst the panelists as they are all considered experts on the research subject.

### Anonymity of the panelists

A study number will be assigned to the panelist to ensure anonymity throughout the procedure. After finishing the study, the panelists will co-publish as collaborator group. Nevertheless, the collected data will not be traceable to the individual panelist. Information on the three rounds, the electronic character of the procedure and publishing as collaborator will be described in the invitation email. Participating is entirely voluntary. Informed consent is implied by participating in the study and panelists are free to withdraw at any time without providing a reason. Hence, given the voluntary and non-invasive character of the study the Human Research Committee was not consulted.

### The Delphi structure

**Fig 1:**
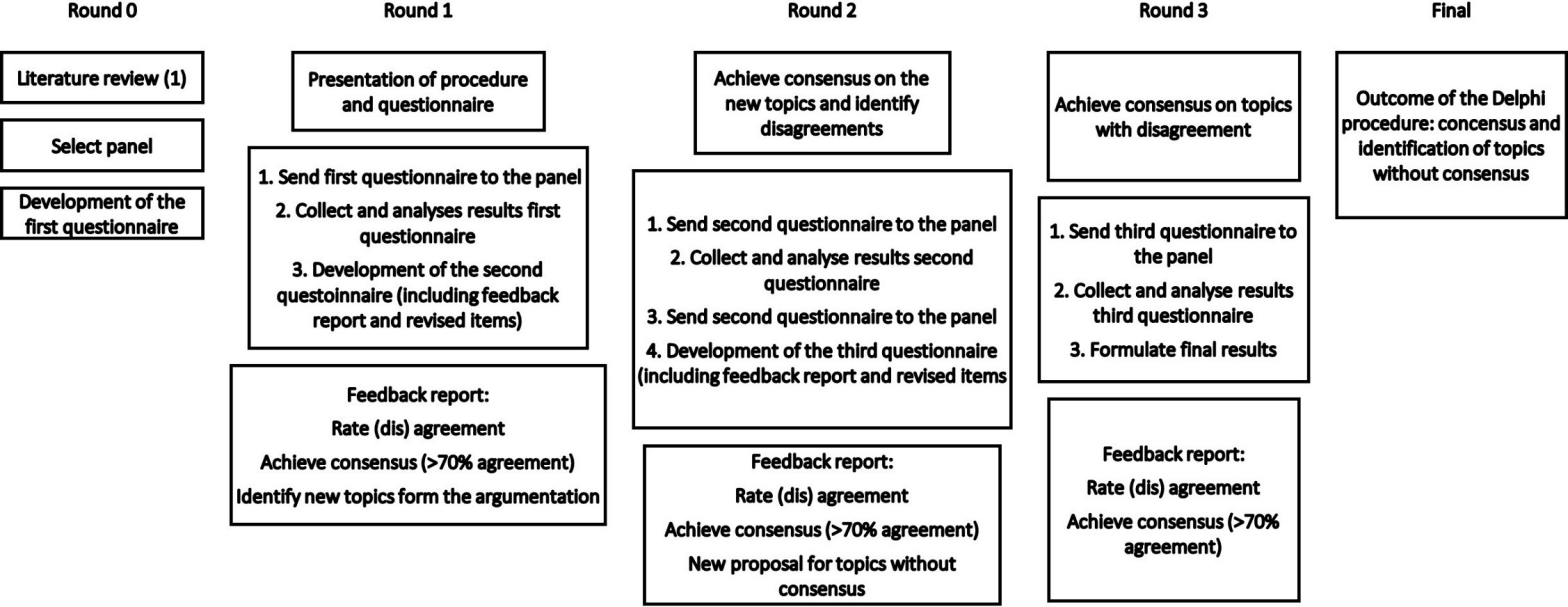
The Delphi procedure on trauma-related preventable death.

A digital three round Delphi procedure will be performed among the participating experts (Fig 1). The most recent literature, including a systematic review, suggests combining a clinical definition and trauma prediction algorithm to form a validated definition of TRPD [3]. However, in the literature there is no consensus amongst experts on the most suitable trauma prediction algorithm [3]. The first round of the Delphi procedure will be used to test the conclusions of the systematic review amongst the panelists. Therefore, the steering committee formed three research objectives:

1) Reaching consensus on the most suitable category out of the four presented categories from the systematic review [3]: I) clinical definition, II) trauma prediction algorithm, III) clinical definition with an additional trauma prediction algorithm and IV) others (e.g., errors in care or extended clinical definition). Ranking most–least suitable.
2) In depth evaluation on the content of the most suitable category.
3) Assessment of the additional benefit of a trauma prediction algorithm. Ranking most–least suitable; Injury Severity Score (ISS), Abbreviated Injury Scale (AIS), Probability of survival (Ps), Revised Trauma Score (RTS), Trauma Injury Severity Score (TRISS).

### Consensus criteria

While consensus is the primary aim of the study, the definition of consensus varies in literature, reporting percentage on agreement between 50–100% [10]. For this study consensus is met in case of 70% or more agreement amongst the panelist [2, 14].

### Closing criteria

For the design of this study a three round electronic survey is decided as a closing criteria by the steering committee. The committee will review the results after each round and provide feedback to the panelists prior to the following questionnaire, to maintain the iterative and controlled feedback character of the procedure.

### Analysis

The questionnaires developed by the steering committee will be distributed using an online survey program Castor EDC (Supplement 2 in S1 File). Data will be collected and managed in Castor EDC, and analyzed using the Statistical Package for the Social Sciences (SPSS) version 25 (SPSS®, Chicago, III, USA).

The collected data will be analyzed by the committee on consensus regarding the four categories and algorithms. Additional argumentation on the multiple-choice questions by the panel members is requested for the in dept discussion and continue with the following round (Fig 1). Hereafter, a feedback report with the results, percentage on agreement and argumentation on the most and least applicable classification and algorithm will be presented to the panelists. This will form the introduction of the second round, where the questions without agreement or with disagreement from the first round will be retested. In addition, new subjects derived from the discussion during the procedure will be addressed and taken into consideration during the following round. This process will be repeated for the third and final round. The collected data and a summary of the argumentation will be published. The remaining data are available upon reasonable request.

## Conclusion

Evaluation of quality of trauma care is of great importance. Hence, the identification and assessment of trauma-related preventable death is essential. Though, the evaluation of quality of trauma care remains suboptimal. As the definition of trauma-related preventable death lacks validity, due to differences in terminology and classification. A Delphi procedure is the assigned technique to reach consensus amongst experts on this subject, in order to establish a widely supported definition on trauma-related preventable death.

## Supporting information

S1 Checklist PRISMA-P 2015 checklist. (DOCX)

S1 File (DOCX)
