# Supplementary material for: Classification of trauma-related preventable death; protocol of a Delphi procedure
Source: PLoS One. 2024 May 6;19(5):e0298692. doi: 10.1371/journal.pone.0298692 (PMC11073670; doi:10.1371/journal.pone.0298692)
Supplement: S1 File — (DOCX) [file pone.0298692.s002.docx]

**Supplement 1.**  Category IV, other (1)

Lu et al. 2020

Non survivable: AIS =6: laceration to the heart, aorta or thoracoabdominal great vessels, massive brain tissue damage or hemorrhage, spinal column dissociation, C1-C3 fracture associated with spinal cord involvement, complete tracheal rupture, fatal chemical exposure and burns with charring.

Potentially survivable: when optimal care had been immediately available.

Smith et al. 2019

Survivable: prehospital care available <10 min of injury and definite care <1 hour (PTLS)

Fatal wounds: involved both cerebral hemi-spheres, the mid-brain or brainstem, cervical spinal cord at or above C5, heart, any non-extremity major vascular structure, or multiple solid organs.

Potentially survivable wounds: vascular structures in the extremities, or torso injuries without the presence of major vascular or multi-visceral injuries.

Drake et al. 2017/2020

TRNP: torso transection, catastrophic brain injury (e.g. brain avulsion, transcranial penetrating or perforating brain injury involving deep nuclei or critical vascular structures, brainstem injury); cervical cord transection (e.g. above cervical level 4); airway transection within the thorax; cardiac injury (e.g. >1/2 inch transmural involvement); thoracic aorta or main pulmonary artery transmural lacerations with free-bleeding; complete hepatic disruption; catastrophic abdominopelvic injury (e.g. lower extremity amputations with open pelvis and large soft tissue loss or traumatic hemipelvectomy); hinge (e.g. transverse fracture of the base of the skull that separates the skull base into 2 halves) or ring (basilar skull fracture around the foramen magnum involving the clivus) skull fractures; facial fractures resulting in transected/fragmented/avulsed trachea/pharynx; compression asphyxia or strangulation; atlanto-occipital disarticulation; and charred remains. Nonpreventable deaths also included patients for whom the primary cause of death was attributed to severe comorbid conditions with contributions from an associated minor injury, provided that the physiological cause of death was not hemorrhage. All patients were adjudicated to have a primary cause of death.

Sarani et al. 2018

TRPPD: pre-hospital care <10minutes of injury and trauma center care <1 hour.

TRNP: wounds involving both cerebral hemispheres, mid-brain or brain stem, cervical spinal cord at or above C5, heart, any non-extremity major vascular structure, or multiple solid organs.

Pot survivable injuries: vascular structures in the extremities, torso injurie without the presence of major vascular or multi-visceral injuries.

Reed Smith et al. 2018

Nonsurvivable injury: penetration of the heart, non-extremity major vasculature structure such as aorta, iliac artery, or mediastinal vessels, bi-hemispheric, midbrain or brainstem injury, transection of the spinal cord above C5, or tracheal disruption.

TRPPD: totality of all wounds would not lead to death had the principles of PHTLS or ITLS <10 minutes and definitive trauma care within 1 hour.

Montmany et al. 2017

Errors: established according to Ps, hemodynamic stability of patients upon arrival to the ER and errors produced by not following the guidelines; taxonomy of JCAHO.

Ha et al. 2016

TRNP: AIS =6 or ≥2: death was judged as NP first, AIS at diagnosis was 5, Ps <50%, upon admission RTS was <6 and their Ps <50%, serious underlying condition (chronic renal failure, liver cirrhosis etc.) or death was due to acute myocardial infarction or pulmonary thromboembolism or patients refused treatment/do not resuscitate.

TRPD: Ps ≥75%

TRPPD: Ps 25-75%

Ray et al. 2016

TRPPD examples: non-devastating traumatic brain injury such as epidural or subdural hemorrhage and hemorrhage from solid organ injuries that were deemed surgically treatable.

TRNP: major rupture of the heart, uncontained laceration or transection of the aorta or thoracic vena cava, massive intraparenchymal brain tissue trauma, atlanto-occipital dislocation or brainstem herniation, and severe charring due to massive burns.

Motomura et al. 2014

TRPD: Ps ≥0.5 and when all staff agreed on survivability with more appropriate pre- and in-hospital care.

TRPPD: discussion regarding the possibility of survival was divided.

TRNP: severe brain injuries or Ps <0.5.

Diamond et al. 2009

TRNP: injuries with an AIS in any body region of 6, an AIS head injury excluding isolated extra-axial hematomas of 5, or an ISS>59.

TRPD: deaths after survivable injury.

Zafarghandi et al. 2003

TRPD: at least four of the panel members agreed on its preventability

TRPPD: three members agreed

TRNP: one or two preventable votes

Stocchetti et al. 1994

TRPD: If the trauma had happened in front of a regional hospital during a normal working day, would the patients have survived? Yes

TRPPD: doubt

Sahdev et al. 1994

TRPD: deaths >1 hour or more after the accident and resulting from hemorrhage into the thorax or abdomen, without attempt at surgical control; intracranial hemorrhage with definite epidural or subdural hematoma and no attempt at surgical evacuation of the clot was evident.

*Probably preventable: Identified areas for performance improvement; more likely than not, death would not have occurred had identified errors been avoided)

**Supplement 2**. Questionnaire; A Delphi procedure on Trauma-related preventable death

Trauma-related death is used as a quality indicator for the evaluation of quality of trauma care. Therefore, it is important to properly asses the trauma-related preventable deaths (TRPD) within the trauma population. In order to be able to do so a well described definition of TRPD is required. However, the definition of TRPD based on current literature lacks validity due to differences in terminology and methodology.

Several steps have been taken to establish a valid definition. First, a systematic review was performed; Hakkenbrak NAG, Mikdad SY, Zuidema WP, Halm JA, Schoonmade LJ, Reijnders UJL, Bloemers FW, Giannakopoulos GF. Preventable death in trauma: A systematic review on definition and classification. Injury. 2021;52(10):2768-777.

Hereafter, a Delphi procedure was constructed, aiming to reach consensus on a valid and clinically applicable definition of TRPD based on the results of the review.

As previously described, the definition of TRPD was divided in four categories. In 42% of the articles a clinical definition was used, in 24% a trauma prediction algorithm was added, sixteen percent solely used a trauma prediction algorithm and in eighteen percent TRPD was defined otherwise. As depicted below.

1. A clinical definition based on panel review or expert opinion:

1. Trauma-related preventable death (TRPD): death could have been prevented if care would have been initiated on time, without avoidable error and/or suboptimal care.
2. Trauma-related potentially preventable death (TRPPD): death could have been prevented given the severity, nonlethal, of the injury under optimal care: timely care, system, evaluation, implementation and treatment generally appropriate; or some deviations from standard of care that could have led to the death.
3. Trauma-related non preventable death (TRNPD): despite adequate care and appropriate management death was unavoidable due to the severity of the injury or comorbidity.

The advantage of using a clinical definition based on expert opinion is based on the collection of relevant data and clinical applicability. However, interobserver variability leads to poor reducibility and reliability, and thus observer bias. Moreover, it might not be suitable for the assessment of a larger number of fatalities or research.

2. Definition based on a trauma prediction parameter or algorithm such as the Injury Severity Score (ISS), Abbreviated Injury Scale (AIS), Probability of survival (Ps), Revised Trauma Score (RTS) or Trauma Injury Severity Score (TRISS).

This definition is more suitable for analysis of larger numbers of fatalities and research due to the well- described algorithms. Patient-related information such as age and comorbidity is not taken into account. Discussion remains on the most suitable algorithm.

3. A clinical definition supplemented with an algorithm, e.g., as stated by the World Health organization and the American College of Surgeons Committee on Trauma:

A. TRPD: death could have been prevented if care would have been implemented on time, without avoidable error or suboptimal care. Ps above 50%, or ISS below 20.

B. TRPPD: death could have been prevented given the severity, nonfatal, of the injury under optimal care: timely care, system, evaluation, implementation and treatment generally appropriate; or some deviations from standard of care that could have led to the death. Ps between 25% and 50%, or ISS between 20 and 50.

C. TRNPD: despite adequate care and appropriate management death was unavoidable due to the severity of the injury or comorbidity. Ps below 25% or ISS above 50%.

D. Non-preventable death, but with care that could have been improved.

The advantage of using this definition is based on the completeness of the clinical definition and reproducibility, due to the addition of a parameter or trauma prediction algorithm. On contrary, the use of the definition might be time consuming and discussion on the most suitable trauma prediction algorithm remains.

4. The fourth category contains the remaining definitions, e.g., based on errors in care or a clinical definition with well-specified injuries (Supplement 2).

This category includes relatively little used definitions. The benefit of using a definition based on errors in care is that improvements can be addressed immediately.

Further to, we would like to pose the following questions:

**Question 1)** Which category is most suitable for the definition of TRPD:

(Please score accordingly, 1. Most suitable – 4. Least suitable)

1. Category I
2. Category II
3. Category III
4. Category IV

Answer:

Argumentation:

**Question 2)** Do you agree in (some) extend with the previously described categories? If not, please provide your suggestion on the definition of TRPD.

1. Yes, I partially agree.
2. Yes, I completely agree.
3. No, I disagree. My suggestion would be to….

Answer/argumentation:

**Question 3)** With algorithm/parameter do you think is most appropriate assessing TRPD?

(Please score accordingly, 1. Most suitable – 5. Least suitable)

1. Injury Severity Score (ISS)
2. Abbreviated Injury Scale (AIS)
3. Probability of survival (Ps)
4. Revised Trauma Score (RTS)
5. Trauma Injury Severity Score (TRISS)
6. Combination of the previously mentioned parameter or other….

Answer:

Argumentation:
